# Chemoprotective effect of arbutin on azoxymethane-induced aberrant crypt foci in rat colon via modulation of PCNA/Bax protein

**DOI:** 10.1590/1414-431X2024e13306

**Published:** 2024-07-01

**Authors:** K.A. Ahmed, S.H. Shareef, T.A. Faraj, M.A. Abdulla, S.K. Najmaldin, N.F.S. Agha, R.K. Kheder

**Affiliations:** 1 Department of Medical Laboratory Sciences, Faculty of Allied Medical Sciences, Al-Ahliyya Amman University, Amman, Jordan; 2 Department of Biology, College of Education, Salahaddin University-Erbil, Erbil, Kurdistan Region, Iraq; 3 Department of Basic Sciences, College of Medicine, Hawler Medical University, Erbil, Iraq; 4 Department of Medical Analysis, Faculty of Applied Science, Tishk International University, Erbil, Kurdistan Region, Iraq; 5 Department of Anesthesia, Erbil Medical Technical Institute, Erbil Polytechnic University, Erbil, Kurdistan Region, Iraq; 6 Medical Laboratory Science Department, College of Science, University of Raparin, Rania, Sulaymaniyah, Iraq

**Keywords:** Arbutin, Azoxymethane, Aberrant crypt foci, Proliferating cell nuclear antigen, Bax protein

## Abstract

Arbutin is utilized in traditional remedies to cure numerous syndromes because of its anti-microbial, antioxidant, and anti-inflammatory properties. This study aimed to evaluate chemopreventive effects of arbutin on azoxymethane (AOM)-induced colon aberrant crypt foci (ACF) in rats. Five groups of rats were used: normal control group (rats injected hypodermically with sterile phosphate-buffered saline once per week for two weeks) and groups 2-5, which were subcutaneously inoculated with 15 mg/kg AOM once a week for two weeks. AOM control and 5-fluorouracil (5-FU) control groups were fed 10% Tween orally daily for 8 weeks using a feeding tube. The treated groups were fed 30 and 60 mg/kg arbutin every day for 2 months. ACF from the AOM control group had aberrant nuclei in addition to multilayered cells and an absence of goblet cells. The negative control group displayed spherical cells and nuclei in basal positions. Histological examination revealed a reduced number of AFC cells from colon tissues of the 5-FU reference group. Arbutin-fed animals showed down-regulation of proliferating cell nuclear antigen (PCNA) and up-regulation of Bax protein compared to AOM control. Rats fed with arbutin displayed a significant increase of superoxide dismutase (SOD) and catalase (CAT) activities in colon tissue homogenates compared to the AOM control group. In conclusion, arbutin showed therapeutic effects against colorectal cancer, explained by its ability to significantly decrease ACF, down-regulate PCNA protein, and up-regulate Bax protein. In addition, arbutin significantly increased SOD and CAT, and decreased malondialdehyde (MDA) levels, which might be due to its anti-proliferative and antioxidant properties.

## Introduction

Cancer is the second leading cause of death in the world, accounting for 9.6 million deaths in 2018 (1). Lung, breast, liver, colorectal, prostate, skin, and gastric cancers are the most common community cancers. Colorectal cancer (CRC) was the most common cause of cancer death worldwide and the third most malignant neoplasm in the world. Colon cancer was created in laboratory animals in numerous studies by various researchers (2-4). Several scientific studies have established countless medicinal plants or their active ingredients for the treatment of colon cancers in laboratory animals (4-7).

Arbutin, a glycosylated hydroquinone, has the effect of an anticancer agent and has cytotoxic properties against several human cancer and tumor cell lines including bladder, bone, brain, breast, cervical, colon, gastric, liver, prostate, and skin cancers (8). Arbutin inhibits osteosarcoma cell proliferation, migration, and invasion via miR-338-3pl MTHFD1L (methylenetetrahydrofolate dehydrogenase (NADP+ Dependent) 1 Like) and by inactivating the AKT (protein kinase B)/mTOR (mammalian target of rapamycin) signaling pathway (8). Previous investigations have documented that arbutin suppresses cell proliferation on MCF-7 and promotes apoptosis in human cancer and melanoma A375 cells (8,9). The repression of aggressive melanoma development by arbutin is associated with the dysregulation of p53, ENOA, and VIME (9).

Arbutin exhibits a range of organic activities, such as antioxidant, anti-microbial, anti-inflammatory, and anti-ulcer effects, and promotes wound healing (10). In addition to its antiproliferative property on osteosarcoma cells (8), Erenler et al. (11) reported that arbutin inhibits HeLa cell proliferation. Arbutin also increases mature neutrophils in murine bone marrow (12). Arbutin is usually used as a skin whitening or depigmenting agent and inhibits tyrosinase, the rate-limiting enzyme of mammalian melanogenesis (13) and, thus, is widely utilized as a skin-lightening cosmetic mediator for the treatment of cutaneous hyperpigmentation disorders, such as melasma and freckles. Moreover, the clinical use of arbutin in humans is safe and effective in decreasing melanin content in addition to tyrosinase action in melanoma cells (13).

Azoxymethane (AOM) is a powerful cancer-causing agent that has been widely used to induce colon cancer in animals. The cytotoxicity of AOM is through oxidative stress. AOM precursors, composite dimethylhydrazine-alkylating mediators, cause DNA transmutation via a necessary methyl or alkyl set of guanine remnants, leading to G to A transmutations (14). AOM, an azomethane oxide, is widely utilized to cause aberrant crypt foci (ACF) in experimental models (4,15), and other studies have used AOM for the induction of colorectal cancer in rats (15,16).

ACF are colon preneoplastic lesions that can be utilized as tools to study defensive methods for CRC. These foci have been used as intermediary biomarkers to promptly assess the chemopreventive effects of several agents, including emerging agents against colon cancer (17). ACF are hyper-proliferative lesions in carcinogen-treated models (4,18). Furthermore, ACF was first recognized as putative pre-cancerous lesions in the colon of carcinogen-treated rats (18) and is known as a colonic carcinogenesis biomarker in addition to other precursors of colon cancer (19).

5-Fluorouracil (5-FU) is one of the most used treatments for CRC and has been extensively utilized in chemotherapy for progressive and metastatic CRC (6,20). 5-FU shows anti-cancer activity via preventing thymidine adenylate synthase action, avoiding methylation of deoxythymidylate, and restoring damaged DNA (21). Many studies by diverse investigators used 5-FU as a reference drug against AOM-induced colon cancer in rats (22,23). The aim of this study was to assess the histopathology, immunohistochemistry, and chemoprotective effects of arbutin against AOM-induced colon cancer in rats.

## Material and Methods

### Chemicals and reagents

Chemicals were bought from Sigma-Aldrich (Switzerland). AOM was diluted in 10 mL of PBS and injected hypodermically in rats at a concentration of 15 mg/kg once a week for two weeks (2). 5-FU was diluted in normal saline and applied as the standard reference medicine preceding intraperitoneal injection to rats at 35 mg/kg for five days (20).

### Experimental design and animals

Adult healthy male rats weighing (150-170 g) were acquired from the Investigational Animal Unit, College of Science, Cihan University, Erbil. Thirty rats were arbitrarily divided into 5 groups with 6 rats in each group. Group 1 received hypodermic inoculations of PBS once a week for 2 weeks and oral gavage of 10% Tween-20 for 2 months. Group 2 was injected with AOM and was fed 10% Tween-20 for 2 months. Group 3 was the positive control and received an intra-peritoneal injection of 35 mg/kg 5-FU for 5 consecutive days. Groups 4 and 5 were fed 30 and 60 mg/kg arbutin, respectively, orally by a feeding tube daily for 2 months.

Groups 2-5 received hypodermal injections of AOM (15 mg/kg) once a week for two weeks. The body mass of rats was measured every week for 2 months. Animals were sacrificed after 24 h of fasting by intramuscular inoculation of ketamine (100 mg/kg) and xylazine (10 mg/kg). Blood specimens were collected for analysis of biochemistry parameters. Colons were rinsed with ice-cold PBS to assess ACF. Colons were opened end-to-end from the anus to the rectum and then placed horizontally between filter paper and fixed with 10% formalin for 30 min at 4°C (24).

### Gross count of ACF in colon epithelium

Colons were cut into three equal portions and then stained with 0.2% methylene blue for 15 min and examined microscopically for ACF counting. Total ACF in the whole colon was calculated, distinguished from neighboring normal crypts by their engorged size, increased space in the basal lamina outside cells, and a noticeable peri-cryptal region (24). The ACF were measured by the quantity of crypts, and each focus was categorized as having four or more than four ACF.

### Histopathological analysis

Colons were cut into 2×2-cm squares and fixed in 10% formalin overnight for histological analysis. Fixed tissue biopsies were sliced and embedded in paraffin wax. Sequential slices of 5-µm thickness were compared to a muscular layer using hematoxylin and eosin. Histopathology of every ACF was used to evaluate crypt nuclear structures compared with normal neighboring crypts (6).

### Immunohistochemical staining

Colon specimen slices of 5-µm thickness in poly-L-lysine slides were placed in an oven at 60°C for 2 h. Slices were dewaxed by xylene, hydrated, and then exposed to antigen recovery by dipping into 10 mM citrate buffer (pH 6) and heating to 92-95°C for 20 min in a microwave. Following the manufacturer’s instructions, the immunostaining stages were completed. Briefly, transient endogenous peroxidase was inhibited by applying 0.03% hydrogen peroxide sodium azide for 5 min. Slices were rinsed with buffer and then incubated with biotinylated primary antibodies against PCNA (1:200) and Bax (1:100) for 15 min (4). Slides were rinsed with washing buffer and preserved in buffer immersion in a moist compartment. An adequate quantity of streptavidin-HRP was added and additional incubation was performed for 30 min. After rinsing, slides were added with diaminobenzidine chromogen substrate and left to develop for more than 7 min, followed by rinsing and counterstaining with hematoxylin for 5 s. Slides were dipped in weak ammonia (0.037 M/L) 10 times, rinsed, and covered with a cover slip. Slides were visualized under light microscopy and appraised by a blinded observer. Positive antigens were stained brown (24). Five slides of the colon from every rat were observed, and 1,500 cells were counted from each region. PCNA staining percent inhibition (PI) was measured as follows: PI = (number of positive cells/total number of mucosal cells) ×100 for every slice. PI was calculated for individual slides and then, the average of the group was calculated.

### Antioxidant and MDA levels in colon homogenate

The colon tissue was homogenized in phosphate-buffered solution utilizing a Teflon homogenizer (Tissue-Tearor, BioSpec Products Inc., USA). The supernatant was discarded, and the sample was centrifuged at 2000 *g* for 10 min at 4°C. The actions of antioxidant enzymes catalase (CAT) and superoxide dismutase (SOD) were measured based on the manufacturer's recommendations. Assessment of oxidative stress in colon homogenates was performed using a commercial thiobarbituric acid reactive substance (TBARS) kit to estimate malondialdehyde (MDA) as a measure of lipid peroxidation (4).

### Ethics statement

The research was approved by the Institute of Animal Maintenance Procedure Board (IACUC), Cihan University-Erbil and Kurdistan Region/Iraq (Ethic No. ERB 85/07/03/2023/MMA). All rats included in the experiments were cared for according to the “Guidelines for the Maintenance and Use of Research Laboratory Animals” issued by the National Institutes of Health (USA).

### Statistical analysis

Data analysis was performed using IBM SPSS statistical software for Microsoft Windows, version 22.0 (SPSS Inc., USA) and GraphPad Prism 9 (USA) was used for histograms. One-way analysis of variance (ANOVA) was used to assess differences between groups with Bonferroni multiple comparisons *post hoc* test. Data are reported as means±SE. The level of significance was set at P<0.05.

## Results

### Macroscopic features and ACF counting

**Figure 1:**
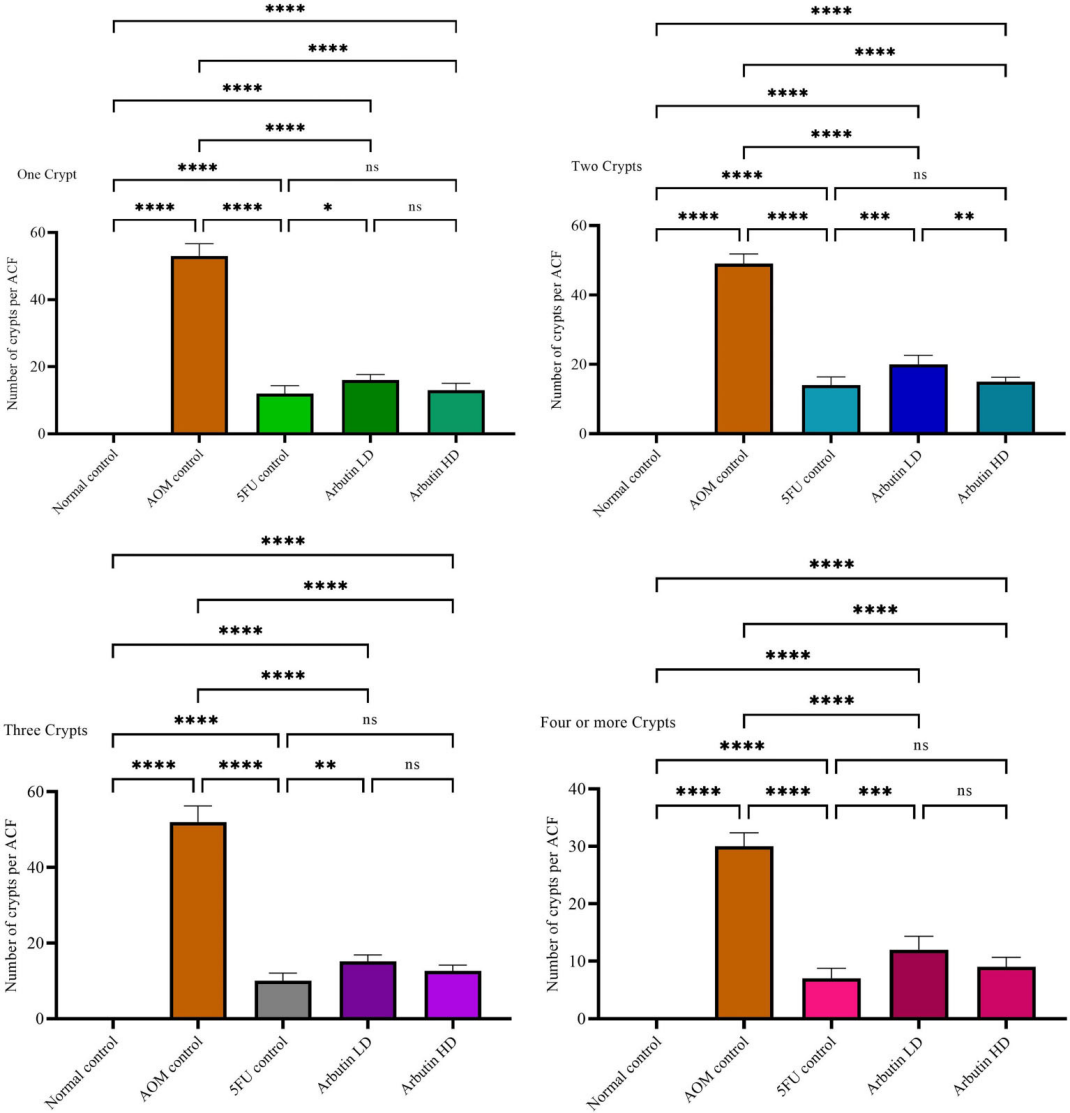
Effects of arbutin on azoxymethane (AOM)-induced aberrant crypt foci (ACF) in the rat colon of animals of the normal control, AOM control, 5-fluorouracil group (5-FU) + AOM, 30 mg/kg arbutin + AOM (LD, low dose), and 60 mg/kg arbutin + AOM (HD, high dose) groups. Data are reported as means±SE for one, two, three, and four or more crypts (n=6). *P<0.05; **P<0.01; ***P<0.001; ****P<0.0001 (ANOVA with Bonferroni). ns: non-significant.

**Figure 2:**
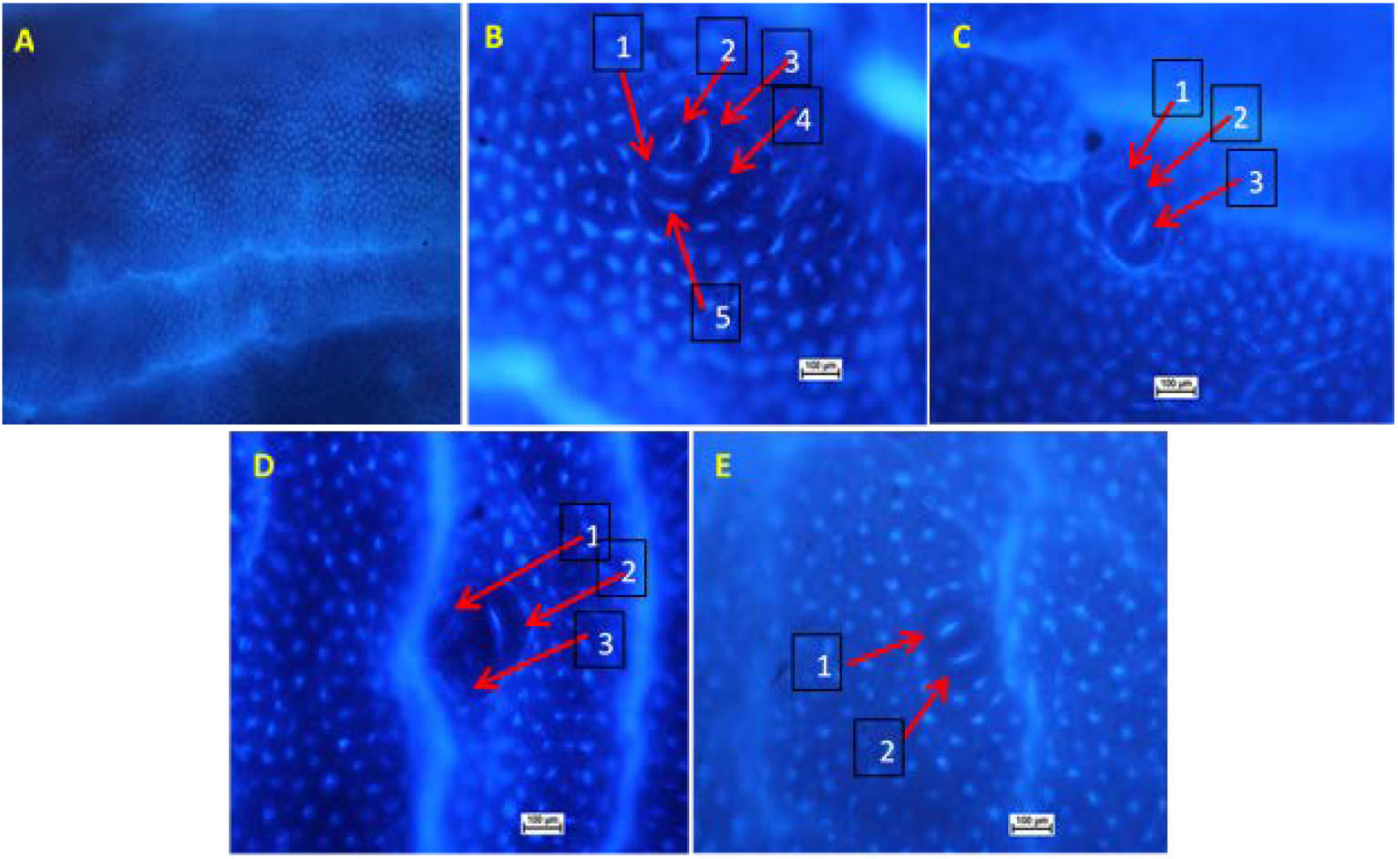
Effects of arbutin on the microscopic appearance of azoxymethane (AOM)-induced aberrant crypt foci (ACF) in rat colon tissue (methylene blue staining, 10×). **A**, Normal control from rats administered 10% Tween 20 showing normal crypts. **B**, AOM group, showing important increases in the number of ACF (arrows). **C**, 5-Fluorouracil group + AOM, showing a decrease in the ACF number (arrows). **D**, 30 mg/kg arbutin + AOM, showing a decrease in the ACF number (arrows). **E**, 60 mg/kg arbutin + AOM, showing a decrease in the ACF number (arrows). Scale bar: 100 µm.

ACF was primarily counted in the mid colon with calculation of the average ACF crypts per focus (Table 1, Figures 1 and 2). As shown in Figure 1, AOM control rats presented recognizable ACFs in the colon. No ACF was observed in untreated colons of normal rats. ACF numbers were considerably greater in AOM control compared to arbutin-fed groups.

**Table 1:** Inhibitory effect of arbutin on azoxymethane (AOM)-induced aberrant crypt foci (ACF) in rat colon of animals of the normal control, azoxymethane (AOM) control, 5-fluorouracil group (5-FU) + AOM, 30 mg/kg arbutin + AOM, and 60 mg/kg arbutin + AOM groups.

Groups	Number of crypts per ACF				Total	Inhibition (%)
	One crypt	Two crypts	Three crypts	Four or more crypts		
Normal control	0	0	0	0	0	0
AOM control	53.0±3.6	49.0±2.8	52.0±42	30±2.3	184±50.7^a^	0
5-FU control	12.0±2.3	14.0±2.3	10.0±2.0	7±1.7	43±8.3^b^	76.6^a^
Arbutin 30 mg/kg	16.0±1.6	20.0±2.6	15.1±1.7	12±2.3	63±8.2^b^	65.7^b^
Arbutin 60 mg/kg	13.0±2.0	15.0±1.2	12.6±1.5	9±1.6	49±6.3^b^	73.3^a^

Data are reported as means±SE (n=6). Data with different superscript letters in the same column are significantly different. ANOVA and Bonferroni's *post hoc* test.

### Histological analysis

**Figure 3:**
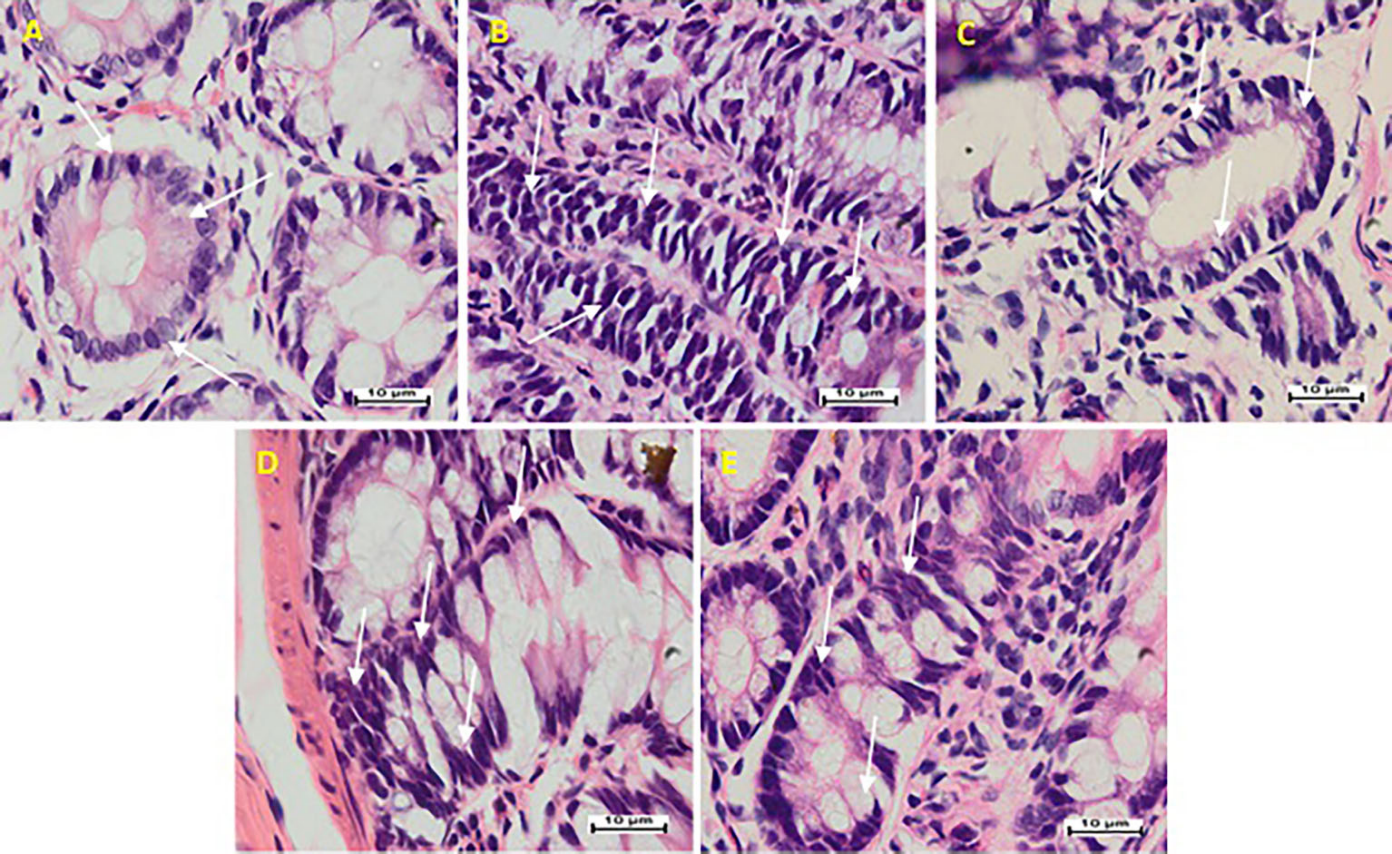
Histological analysis of the effect of arbutin on azoxymethane (AOM)-induced aberrant crypt foci (ACF). Segments were cut adjacent to the muscular stratum and stained with HE (100×, scale bar: 10 µm). **A**, Normal colon epithelia displaying round shaped crypts with nuclei with basal polarization and no stratification (arrows). **B**, AOM group with elongated ACF and a small lumen, noticeable nuclear atypia (elongated and stratified nucleus), basal nuclear polarization, and reduced goblet cells (arrows). **C**, 5-Fluorouracil group + AOM with slightly elongated ACF and nuclei, slightly fewer goblet cells, and no nuclear stratification (arrows). **D**, 30 mg/kg arbutin + AOM with elongated ACF and nuclei, minor reduction in goblet cells, and nuclei with mild stratification (arrows). **E**, 60 mg/kg arbutin + AOM with ACF with a somewhat round contour and no stratified nuclei, slightly reduced goblet cells, and proliferation nuclei (arrows).

The tissue from the normal control group displayed rounded cells with the nucleus in basal position. AOM control comprised increased ACF, showing obvious nuclear atypia, reduced goblet cells, decreased mucin, and smaller lumen. The length of ACFs was greater than that of normal crypts, and the cytoplasm was strongly stained. Mucosal cells in ACF had enlarged and faintly stratified nuclei, damaged cell polarization, and increased mitosis compared to adjacent normal crypts (Figure 3). Arbutin-fed groups displayed a rise in apoptosis, condensed mitosis, cellular propagation, and abridged initiation of ACF, with minor morphology alterations compared with the AOM control group.

### Immunohistochemistry findings

**Figure 4:**
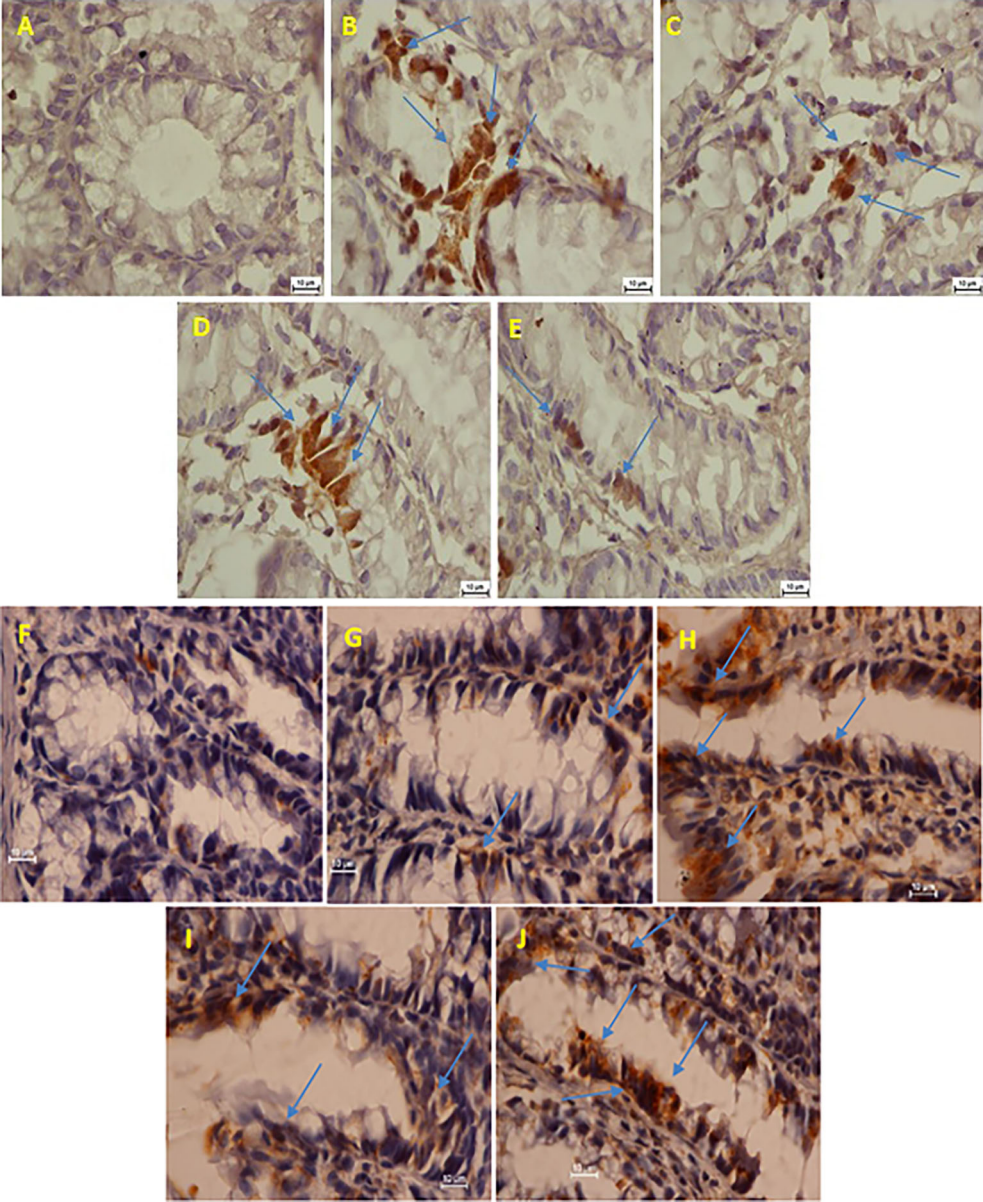
Effects of arbutin on immunohistochemistry staining of proliferating cell nuclear antigen (PCNA) in azoxymethane (AOM)-induced colon cancer in rats. **A**, Normal control group with no PCNA staining. **B**, AOM-control group with up-regulation of PCNA. **C**, 5-Fluorouracil-fed + AOM group with down-regulation of PCNA. **D**, Animals fed 30 mg/kg arbutin + AOM showing down-regulation of PCNA. **E**, Animals fed 60 mg/kg arbutin + AO, showing down-regulation of PCNA. **F**-**J**, Effects of arbutin on immunohistochemistry staining of Bax proteins in AOM-induced colon cancer in rats showing down-regulation of Bax in animals fed 5-FU or arbutin. Blue arrows indicate the down-regulation of Bax proteins. Magnification 100×, scale bar: 10 µm.

Arbutin induced the down-regulation of PCNA protein. PCNA was assessed as an indicator of cellular proliferation in colon samples. Slices representative of the groups are presented in Figure 4A-E. PCNA staining intensity (brown, red) of cell nuclei of AOM control rats were more abundant and stronger than arbutin-fed rats.

### Arbutin-induced up-regulation of Bax protein

In the AOM control group, colon slices had significantly weaker immunohistochemistry staining for Bax proteins. A significant increase in Bax protein staining was noticed in arbutin-fed groups (Figure 4F-J).

### Arbutin increased the activities of enzymatic antioxidants

**Figure 5:**
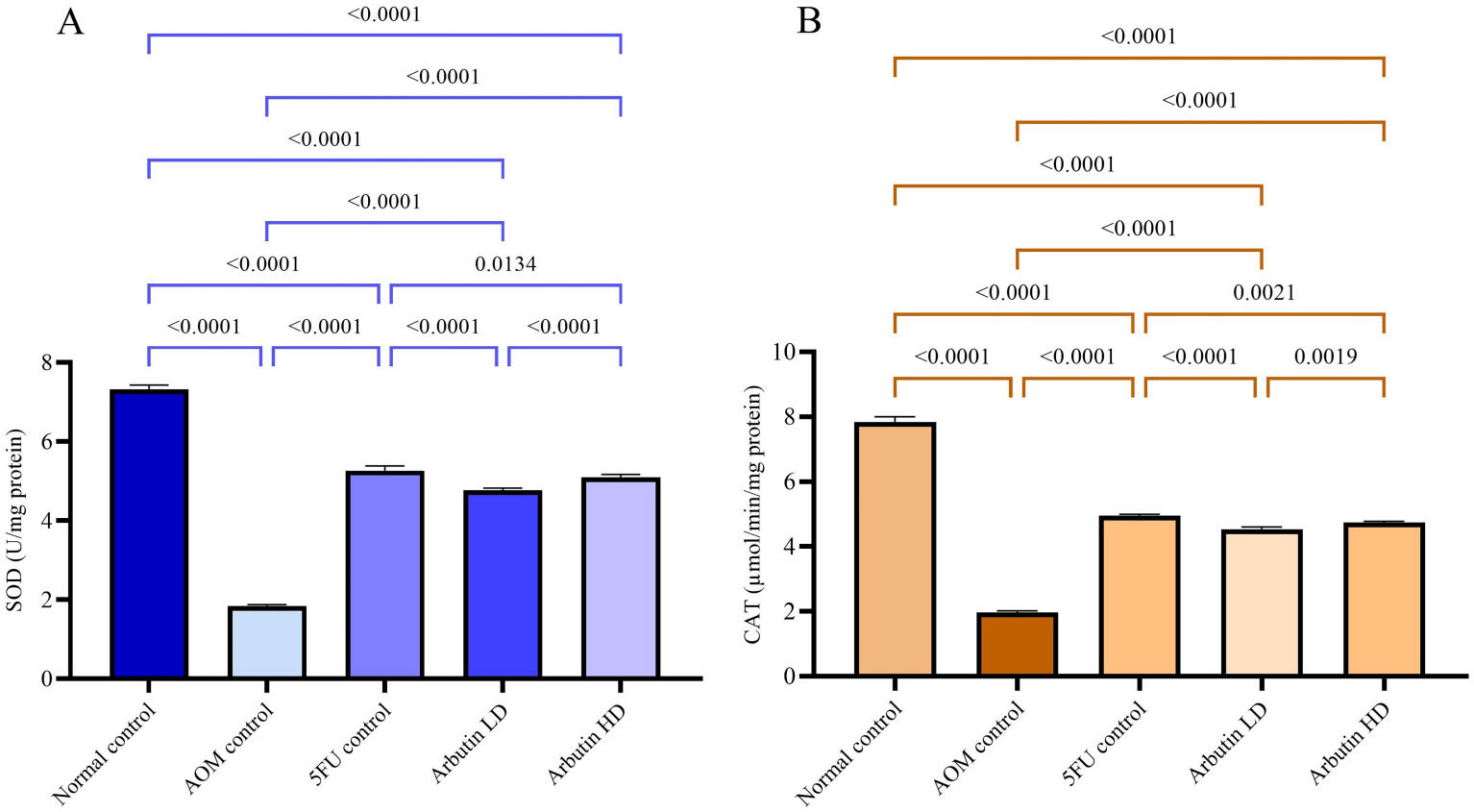
Effect of arbutin on superoxide dismutase (SOD) (**A**) and catalase (CAT) (**B**) activities in colon tissue homogenate of animals of the normal control, azoxymethane (AOM) control, 5-fluorouracil group (5-FU) + AOM, 30 mg/kg arbutin + AOM (LD, low dose), and 60 mg/kg arbutin + AOM (HD, high dose) groups. Data are reported as means±SE. P<0.05 (ANOVA with Bonferroni).

Rats injected with AOM and fed with arbutin or 5-FU had significantly improved SOD and CAT activities in tissue homogenate compared to AOM control group. The arbutin-fed groups had significantly increased enzymatic activity that was restored to near normal levels (Figure 5A and B).

### Arbutin suppressed MDA levels in AOM-induced ACF in rats

**Figure 6:**
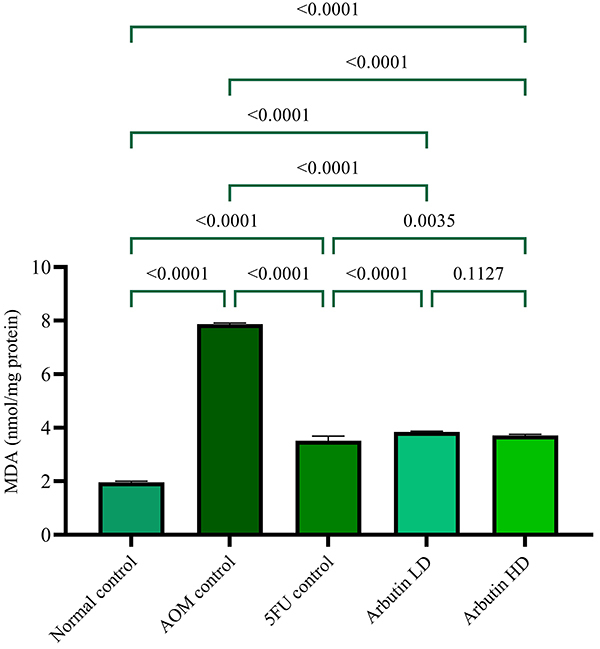
Effect of arbutin on malondialdehyde (MDA) in azoxymethane (AOM)-induced aberrant crypt foci (ACF) in tissue homogenate of animals of the normal control, azoxymethane (AOM) control, 5-fluorouracil group (5-FU) + AOM, 30 mg/kg arbutin + AOM (LD, low dose), and 60 mg/kg arbutin + AOM (HD, high dose) groups. Data are reported as means±SE. P<0.05 (ANOVA with Bonferroni).

Rats fed with arbutin had lower near-normal MDA levels compared to the AOM control group. Accordingly, the aggressiveness of AOM was decreased in rats that received either arbutin or 5-FU (Figure 6).

## Discussion

The presence of ACF was used as one of the indicators of colon neoplasia to evaluate the chemopreventive effect of arbutin in experimental animals. ACF were found to be progressive in the AOM control group in contrast to the normal group, as demonstrated by diverse analyses of crypts with greater dimensions and increased luminal mucosa. Also, the number of crypts was greater in the AOM control group compared to rats fed with arbutin. Similarly, the increase in the number of ACF in the AOM control group was previously reported by various investigators (4,19,25). Rats fed with arbutin displayed no substantial alteration compared with the control rats. Comparable outcomes were found by other investigators (26). The middle part of the colons presented the greatest accumulation of ACF, similar to previous studies (2,19).

In the AOM control group, the histology of ACF showed cells with elongated and stratified nucleus and reduction of mucosal glands compared to arbutin-fed rats. The amount of mucin discharged from mucosal glands could be directly linked to ACF, due to significant and deep colon wall injury. Arbutin could prevent cellular disturbance and deformity, as it conserved colon morphology and then contributed to mucosal protection. The results of the present study are in line with numerous previous studies (18,27,28).

Unrestrained tumor cell propagation has long been revealed to play a critical part in colon carcinogenesis. PCNA, a cofactor of DNA polymerase, is necessary for cell proliferation and has a significant role in the increase of numerous cancers, including CRC (29). Arbutin treatment augmented the expression of Bax proteins and reduced expression of PCNA proteins, which could lead to activation of the immune system, which might be part of the mechanism by which arbutin inhibits AOM-induced cell proliferation (ACF) in colonic tissue. The finding of the present study also showed that PCNA was significantly down-regulated in arbutin-fed rats compared to the AOM control group. Similar findings were previously described by other researchers (24,30). In addition, several investigators using various medicinal plant extracts or their active constituents showed down-regulation of PCNA protein and up-regulation of Bax protein (24,31,32).

Apoptosis is a process essential for normal development and homeostasis in multicellular organisms and important in controlling cell number and proliferation (33). However, disruption of the balance between cell proliferation and apoptosis is one of the main biological events that favors the carcinogenesis process. Activation of apoptotic pathways is a key mechanism by which cytotoxic drugs kill cancer cells. Therefore, the apoptotic pathway is widely studied as a potential target for cancer chemotherapy (5). The reduction in cell proliferation and the increase in apoptosis in colonic tissues by arbutin seem to be related to the reduction in aberrant crypt multiplicity.

Bax, as the pro-apoptotic protein found in the outer membrane of mitochondria, regulates cell life and death through the induction of apoptosis by controlling the penetrability of the mitochondria membranes (28). Bax protein has been measured as an inducer of apoptosis by dimerization and translocation into the outer membrane of the mitochondria, producing a route for additional protein secretion, mainly cytochrome C, but this pathway could be reversed by Bcl-2 (33). Therefore, the balance of the expressions of these proteins is vital for proper cellular function and any change in this balance will directly change the mitochondrial apoptosis pathway (28). Moreover, researchers have shown that the activation of the apoptotic factors caspase-8 and caspase-10 (inducers of the extrinsic pathway) and caspase-9 (inducers of the intrinsic pathway) are engaged in the activation of caspase-3 and apoptosis (33).

The elimination of ROS is vital for cell homeostasis, and any disruption or absence of ROS control could lead to severe cell injury, decreasing normal function, altering gene expression and cell metabolism, and leading to cell death (34). The present research showed the effect of arbutin in balancing ROS level. This biological action could be due to the scavenging of free radicals and prevention of oxidative stress, which avoids colon tissue impairment and promotes the colon tissue healing pathway. Oxidative stress is one of the central factors of inflammation, which is mostly due to uncontrolled ROS production that decreases antioxidant enzymes essential to the removal of free radicals (34). ROS can have a regulatory influence on many apoptotic (caspases, Bax) or anti-apoptotic proteins. Augmented ROS production has been associated with elevated expression of anti-apoptotic proteins and apoptotic irregularities, subsequently causing various pathological diseases including cancer. ROS can cause cellular damage through the oxidation of several essential molecules such as proteins, lipids, or DNA (34).

Arbutin supplementation produced an important positive modulation of Bax protein in rat colon, which could be the molecular mechanism that lowered ACF occurrence leading to the chemoprotective action in AOM-pre-treated rats. Our results were similar to previous research utilizing medicinal plant extracts or their active constituents that reported up-regulation of Bax protein and down-regulation of ACF in AOM-induced colon cancer in rats (16). It has been previously reported that the presence of Bax in colon cancer cells can aggravate cancer in laboratory animals (20). In contrast, a decrease in Bax has a positive influence on cancer development (23).

Antioxidant enzymes such as SOD and CAT eradicate the actions of free radicals in the organism (27). SOD is an enzyme that scavenges peroxide anion radicals preventing lipid peroxidation by free radicals (27). Arbutin administration was found to increase the activities of these enzymes. Similarly, Shwter et al. (32) reported that administering AOM led to a reduction in SOD and CAT enzyme activity levels and increased MDA levels by negatively affecting the functioning of these enzymes. Accordingly, they stated that AOM induced ACF and caused oxidative stress (18).

The 5-FU reference standard drug group presented an analogous outcome to the arbutin-fed group. Our findings indicated that arbutin-fed animals significantly decreased MDA levels and increased SOD and CAT activities in rat tissue homogenate compared to the AOM control group. Similar results have been reported by numerous investigators (24,27,35).

### Conclusion

The current research could be considered the first investigation describing a chemopreventive effect of arbutin against AOM-induced colorectal cancer via reducing the number of ACFs by an anti-proliferative action. Arbutin prevented cancer also by down-regulating PCNA and up-regulating the expression of the Bax protein. It also increased the antioxidant enzyme activities and lowered MDA levels, protecting colon tissues from oxidative damage caused by AOM.
